# Analyzing physiological signals recorded with a wearable sensor across the menstrual cycle using circular statistics

**DOI:** 10.3389/fnetp.2023.1227228

**Published:** 2023-10-19

**Authors:** Krystal Sides, Grentina Kilungeja, Matthew Tapia, Patrick Kreidl, Benjamin H. Brinkmann, Mona Nasseri

**Affiliations:** ^1^ School of Engineering, University of North Florida, Jacksonville, FL, United States; ^2^ Bioelectronics Neurophysiology and Engineering Laboratory, Department of Neurology, Mayo Clinic, Rochester, MN, United States

**Keywords:** menstrual cycles, circular statistical analysis, physiological signal processing, autoregressive integrated moving average, wearable sensor, follicular phase, luteal phase, ovulating/non-ovulating

## Abstract

This study aims to identify the most significant features in physiological signals representing a biphasic pattern in the menstrual cycle using circular statistics which is an appropriate analytic method for the interpretation of data with a periodic nature. The results can be used empirically to determine menstrual phases. A non-uniform pattern was observed in ovulating subjects, with a significant periodicity (p < 0.05) in mean temperature, heart rate (HR), Inter-beat Interval (IBI), mean tonic component of Electrodermal Activity (EDA), and signal magnitude area (SMA) of the EDA phasic component in the frequency domain. In contrast, non-ovulating cycles displayed a more uniform distribution (p > 0.05). There was a significant difference between ovulating and non-ovulating cycles (p < 0.05) in temperature, IBI, and EDA but not in mean HR. Selected features were used in training an Autoregressive Integrated Moving Average (ARIMA) model, using data from at least one cycle of a subject, to predict the behavior of the signal in the last cycle. By iteratively retraining the algorithm on a per-day basis, the mean temperature, HR, IBI and EDA tonic values of the next day were predicted with root mean square error (RMSE) of 0.13 ± 0.07 (C°), 1.31 ± 0.34 (bpm), 0.016 ± 0.005 (s) and 0.17 ± 0.17 (*μ*S), respectively.

## 1 Introduction

**FIGURE 1:**
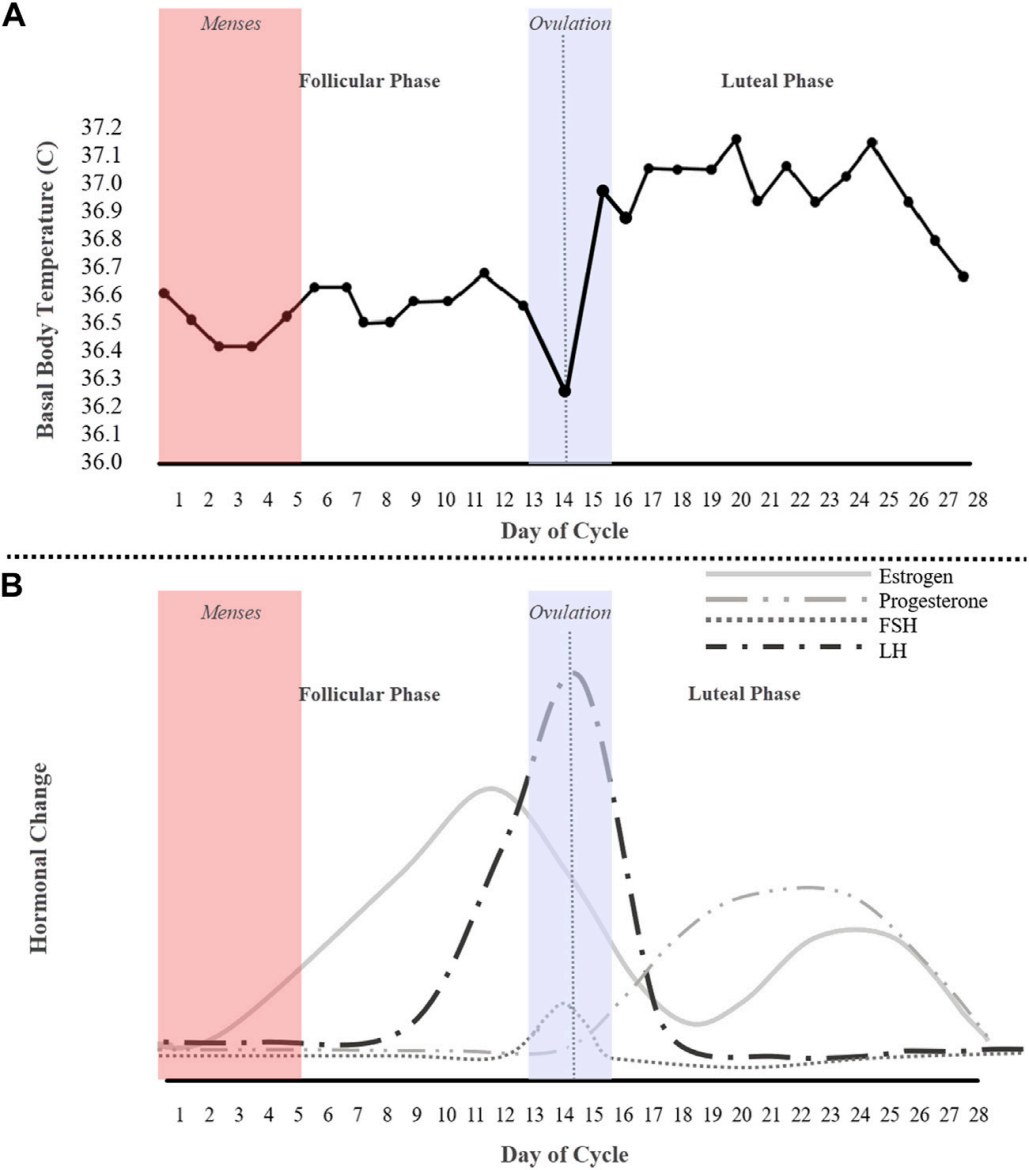
An idealized 28-day cycle illustration of **(A)** a basal body temperature (BBT) chart of an ovulating subject depicting a biphasic pattern, and **(B)** fluctuations of the ovarian hormones estrogen, progesterone, follicle stimulating hormone (FSH), and the luteinizing hormone (LH).

Among methods that have been used to study the menstrual cycle to detect ovulation, such as transvaginal ultrasonography, and cervical mucus inspection, the most common, and less invasive methods, are basal body temperature (BBT) tracking and/or LH-testing (Luteinizing Hormone). In normal physiology, the follicle stimulating hormone (FSH) and luteinizing hormone (LH) activate the ovary and produce follicles. The FSH motivates the growth of ovarian follicles and, of the 30–40 developing follicles, typically only one is released per month (Schmalenberger et al., 2021; Kiranmai and Lakshmi, 2021). The mature follicle then produces increasing amounts of estrogen, resulting in the LH wave (Figure 1A). Traditionally, LH-testing is used to determine if a menstrual cycle is ovulatory and when ovulation occurs. The urinary LH kit has been found to be more accurate than BBT tracking; however, a BBT chart (Figure 1B) can determine the onset of ovulation (San Roman et al., 1995). This method is often the most popular and cost-effective for predicting ovulation, but not the most accurate and oftentimes procedurally daunting—the BBT must be measured every morning before the first urine with a thermometer (Bull et al., 2019; Coyne et al., 2000). As depicted in Figure 1A, BBT is low across the follicular phase, dips before ovulation, increases sharply at ovulation, and then remains elevated across the luteal phase. Essentially, a BBT provides information on the existence of either a biphasic or monophasic pattern, where a biphasic BBT is indicative of ovulation.

Another technique to determine the ovulation day, or fertile window, is the forward/backward-count method (Schmalenberger et al., 2021), which estimates the ovulation day as the middle of the cycle. However, this technique depends on the cycle length. In recent years, this technique has been implemented in smart-phone applications to track menstrual cycles and predict the ovulation day, or fertile window. The fertile range, however, varies broadly even with the suggested “most fertile” days and most of the publicly-available applications are inaccurate (Setton et al., 2016). Thus, tracking ovulation requires additional methodologies to improve reliability, which this study aims to do through a combinatorial approach including analyzing physiological signals, self-reported urine test, and self-reported menses.

Few adopted methods using a combination of physiological signals have been utilized to predict ovulation (Schmalenberger et al., 2021). Signal processing techniques can be used to predict the fertile window more accurately and avoid burdensome traditional methods like LH-testing. Studies have demonstrated that signals recorded continuously with wearable sensors, such as heart rate (HR), skin temperature, and heart rate variability (HRV), respond to different phases of the menstrual cycle (Tenan et al., 2014; Alzueta et al., 2022; Schmalenberger et al., 2021; Yu et al., 2022). In particular, Schmalenberger et al. (2020) showed that HRV decreases significantly from the follicular to the luteal phase in naturally cycling subjects. Authors in (Vishrutha et al., 2012) concluded that the high frequency, or HF (0.15–0.40 Hz) component of HRV was higher in the follicular phase, while the low frequency, or LF (0.04–0.15 Hz) component, was found to be higher in the ovulatory and luteal phases.

To investigate whether physiological parameters are changing across the menstrual cycle, regression models have been used widely in literature (Shilaih et al., 2018). For example, changes in physiological signals such as temperature, HR and HRV recorded from the Oura ring (Ōura Health Oy, Oulu, Finland) across the four menstrual cycle phases (Menses, Ovulation, Mid-luteal and Late-luteal) were statistically tested using Hierarchical Linear Regression models in (Alzueta et al., 2022). It was concluded that there were phase-based shifts in nightly skin temperature and HR across the four phases. Another study (Goodale et al., 2019) considering a series of multilevel models with random slopes and random intercepts, demonstrated that wearable technology (Ava bracelet, Ava, Zurich, Switzerland) can detect concurrent phase-based shifts in wrist temperature, HR and respiratory rate (p < 0.001). The authors also developed an ensemble tree-based machine learning method to separate three main classes: follicular phase, fertile window and luteal phase. This resulted in an overall F-score of 0.78 and it detected a 6-day fertile window in cycles with a 90% accuracy. The classifier was trained on 11 features extracted from the physiological signals, including HR, breathing rate, wrist skin temperature and HRV. The training and testing data split was performed randomly with a 75:25 ratio.

Although many research studies focus solely on subjects with “regular” cycles (with a length of 25–35 days), Yu et al. (2022) has shown that BBT and HR were significantly higher during fertile phase than the follicular phase and peaked in the luteal phase (p < 0.001) in both regular and irregular cycles ( < 25, > 35 days). In Yu et al. (2022) linear mixed models were used to assess changes in physiological signals and a probability function estimation model was developed to predict the fertile window and menses.

In this study, to examine the changes in physiological features during menstrual cycles, circular statistics were exploited. Circular statistics, known as directional statistics, are generally utilized in applications using data in the *R* ^2^ plane, not a linear scale (R) (Pewsey and García-Portugués, 2021; Landler et al., 2019), considering the periodic nature of angular measurements, such as angles, directions, or times. Circular statistics were developed first in the late 19th and 20th centuries. However, they gained more attention in the mid-20th century (Fisher, 1922), and during the latter half of the 20th century, circular statistics found applications in various fields, including biology, once it was realized that circular data was prevalent in many natural phenomena and human activities such as biological rhythms, animal migration patterns, and wind directions.

Recently, circular statistics have become more accessible and widely used due to the advancement of computational power and statistical software. Many sophisticated techniques now exist for analyzing and interpreting circular data, including circular-linear regression, circular mixed-effects models, circular clustering methods, and circular data visualization tools (Cremers and Klugkist, 2018). Circular statistics have been utilized in many fields of research including neuronal activity and other biological researches such as immunology (Cremers and Klugkist, 2018; Karoly et al., 2021; Landler et al., 2019; Gregg et al., 2023). In this paper, circular statistics is used to show periodicity in physiological signals across a natural cycle by analyzing the changes in signal/feature amplitude.

Furthermore, different methods were introduced to test for a deviation from uniformity in circular data, such as the Rayleigh, Rao’s spacing, V, Omnibus and Watson-Williams tests. Note that this study focuses primarily on the Rayleigh test, which evaluates periodicity and non-uniform patterns in data. Additionally, the Watson-William’s test was used to compare ovulating and non-ovulating cycles. The alternative options (Rao, Omnibus, and V tests) were also examined for a comparative discussion.

In this study, the conventional LH-testing method for tracking ovulation was combined with that of previously mentioned physiological signals (HR, HRV and skin temperature) in addition to the electrodermal activity (EDA), among ovulating and non-ovulating women. Several features from temperature, HR, inter-beat interval (IBI), and EDA were extracted. The features to best represent a biphasic pattern (identifiable in ovulating cycles) were selected for the subsequent circular statistical analysis. Finally, we also report accuracies for predicting physiological signals using an Autoregressive Integrated Moving Average (ARIMA) model, which is, in essence, a regression algorithm specifically tailored for time series forecasting. Throughout the text, the cycles are labeled as either ovulating or non-ovulating based on whether the subject’s LH test during that cycle showed positive or negative, respectively.

The paper is organized as follows: Materials and methods provides detail on data collection and processing techniques, as well as, analytical methods, including circular statistics, and ARIMA modeling. In the Results section, features with a noticeable non-uniform pattern are determined, and a comparison between ovulating and non-ovulating cycles has been performed. Finally, the Discussion and Conclusion sections are presented.

## 2 Materials and methods

To examine the relationship between changes in physiological signals, and menstrual cycles, fifteen healthy female individuals were recruited to wear a research-grade wearable wristband (two to four menstrual cycles) while tracking their ovulation. Collected data was processed, and the best features that show deviation from uniformity in ovulating subjects was determined using circular statistic test, Rayleigh. Additionally, an ARIMA model was designed to predict physiological data for the following day. The following sub-sections describe in detail the subject recruitment process, physiological data, and data analysis techniques.

### 2.1 Ambulatory data collection

The Empatica E4 wristband (Empatica Inc., Boston MA) was used to continuously collect physiological data across the menstrual cycles. An Institutional Review Board protocol (IRB 18-00628) has been approved to collect data from female subjects (18–40 years old) at the University of North Florida. Fifteen subjects were recruited to wear the wristband for at least one menstrual cycle, during which each subject marked their menstruation and ovulation days on a calendar. To detect ovulation, each subject was asked to utilize urine test strips, starting the day after menstruation for 2 weeks, or until the test strip depicted a positive result. Subjects who had history of pregnancy within 6 months prior, breastfeeding, working at night shifts, frequently flying across time zones or experiencing sleep disorders were excluded from the study.

In total, data was collected from 46 cycles. Removed were cycles missing more than 5 consecutive days (5 cycles) as well as cycles in which the subject had major sickness (2 cycles) that could affect temperature and heart rate, resulting in statistical analysis of 39 cycles in total; 31 of these cycles had confirmed ovulation, while 8 cycles were absent of ovulation. Non-ovulating subjects were on a birth control, however, they were asked to use the urine test to confirm this. A demographic breakdown of the participating subjects can be found in Table 1.

**TABLE 1:** Demographic information and characteristics of participants included in the analysis.

	Ovulating	Non-ovulating
	(N = 12, 31 cycles)	(N = 3, 8 cycles)
Age, mean	24.25 ± 2.93	24.33 ± 2.31
Age groups, years		
18–24	8	2
25–30	3	1
31–35	1	
BMI, mean (*lb*/*in* ^2^)	27.07 ± 4.47	25.744 ± 1.48
BMI groups		
<18.5		
18.5–24.9	6	1
25.0–29.9	3	2
≥30.0	3	
Duration of menstruation, mean (days)	5.48 ± 0.78	5.13 ± 1.25
Duration of cycle, mean (days)	28.87 ± 4.19	28.36 ± 1.77

### 2.2 Physiological signals

The Empatica E4 Wristband collects several physiological signals: electrodermal activity (EDA), temperature, inter-beat-interval (IBI), accelerometer, and blood volume pulse (BVP). The Blood Volume Pulse (BVP) is the primary output from the PPG data (Torres et al., 2016), which is obtained by optical sensors (Fortino and Giampà, 2010). HR and IBI are derived from BVP, where IBI is the time interval between individual heartbeats and is computed from detecting peaks of the BVP and its variability defines the HRV (Shaffer and Ginsberg, 2017). Statistical features were extracted from each individual signal. From IBI, autocorrelation, LF and HF powers in frequency bands of 0.04–0.15 Hz, and 0.15–0.4 Hz respectively, and normalized LF power were calculated (Tenan et al., 2014).

Electrodermal Activity (EDA), usually measured in *μ*S (micro Siemens), refers to the electrical changes measured on the epidermis, which arises when it receives innervating signals, such as stress, from the brain (Benedek and Kaernbach, 2010; Djawad et al., 2019; Shukla et al., 2019; Zangróniz et al., 2017). By applying a low voltage, EDA sensors can non-invasively measure skin conductance (Djawad et al., 2019). The skin conductance is characterized into two types: Tonic Skin Conductance Level (SCL), and rapid Phasic Skin Conductance Response (SCR) (Braithwaite et al., 2013). The Tonic SCL is generally considered to be the slowly varying component of skin conductance. The other component, SCR, is the faster changing element (Braithwaite et al., 2013; Benedek and Kaernbach, 2010) and represented by bursts of peaks. Numerous studies (e.g. (Zangróniz et al., 2017; Benedek and Kaernbach, 2010; Egan et al., 2016)) have shown that there is a direct correlation between physiological signals, such as heart rate, and EDA.

From EDA, statistical features, area under both phasic and tonic components, signal magnitude area (SMA) in both frequency and time domains, SCR peak count, SCR peak width as well as normalized power in frequency ranges between 0.1 and 0.5 in steps of 0.1, were calculated. The SMA in frequency domain, was calculated by taking the sum of the absolute value of the Fourier Transform of the phasic component.

### 2.3 Minimizing artifacts

The challenging aspect with wearable devices is the quality of the data recorded, which becomes more important in ambulatory settings and long-term data collection. Several algorithms were introduced to remove data epochs corrupted by artifacts using signal quality metrics (Böttcher et al., 2022; Nasseri et al., 2020). It was shown that the quality of the signals were higher at night than during the day (Böttcher et al., 2022), because motion artifacts are the main source of data corruption.

Therefore, some studies focus only on analyzing data recorded during the night, or sleep times. In Shilaih et al. (2018) subjects were asked to wear an Ava bracelet during sleep to continuously record wrist skin temperature (WST). The Ava bracelet provided one measurement every 10-s. To avoid variation in temperature induced by the sleep onset and waking up, the first 90 and the last 30-min of each night’s data were excluded. To remove artificial fluctuations, the temperature signal was smoothed before statistical analysis. In another study, nocturnal data was collected using the Oura ring from 10-pm to 8-am. To remove the data fluctuations, a moving average filter with a length of 17-min was applied to data (Maijala et al., 2019).

Although data in this study was collected from subjects wearing the device the whole time, to minimize the effects of artifacts, sleep data was extracted during no-movement (or minimum movement) hours by implementing a simple algorithm to detect changes in hand angle using accelerometry data (van Hees et al., 2018). A threshold was determined to detect the no-movement hours, within which actual sleep hours were identified via HR thresholding. The next section describes the sleep data extraction in more detail.

### 2.4 Data preprocessing

**FIGURE 2:**
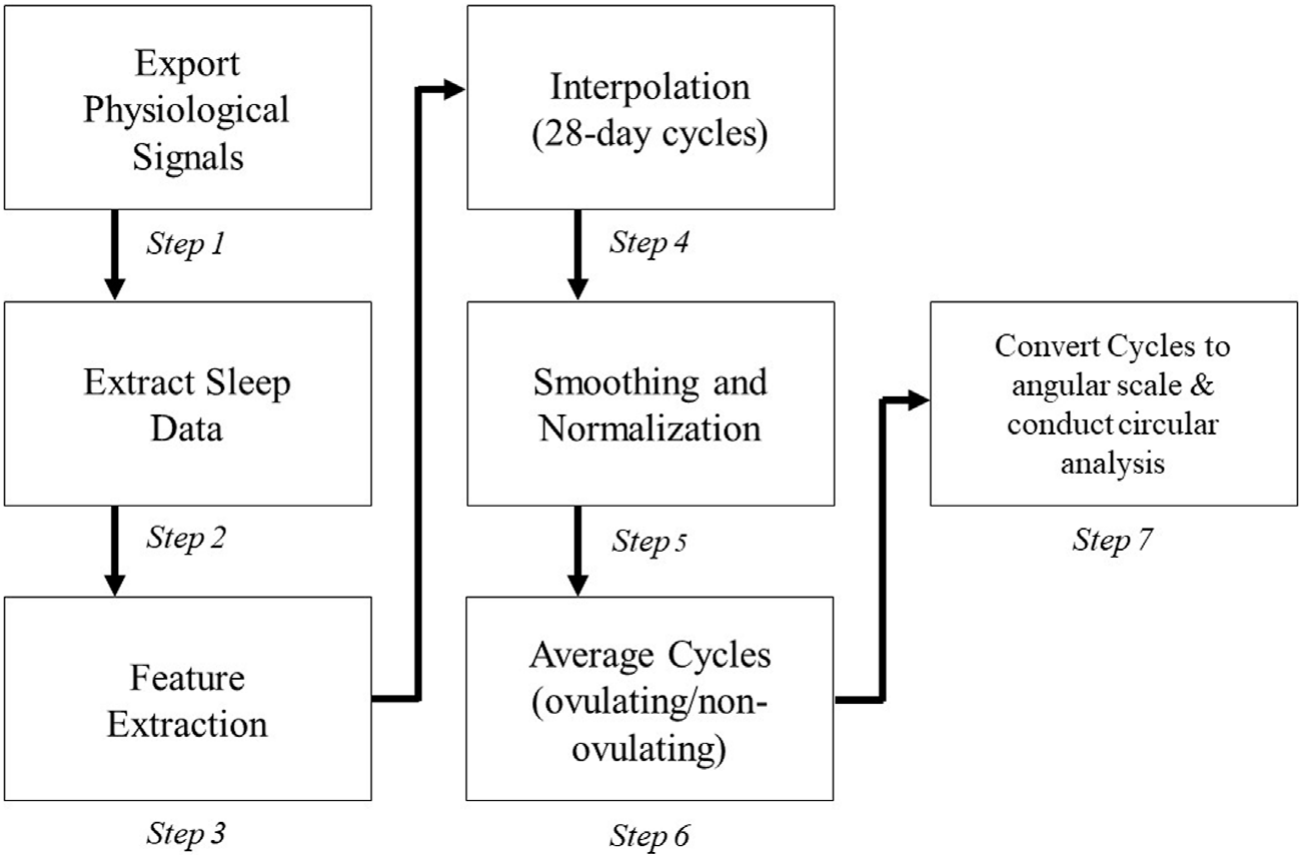
Steps of the heuristic algorithm for processing and plotting each physiological signal.

The heuristic algorithm applied to each subject’s cycle is illustrated in Figure 2 after removing cycles missing more than 5 days or cycles in which the subject had a major sickness. There is variability among what a regular menstrual cycle length range should be; however, many studies fall in the range from 25–35 days for healthy pre-menopausal women (Campbell et al., 2021; Schmalenberger et al., 2021; Fehring et al., 2006). The cycles outside of this range with a positive luteinizing hormone test (indicating ovulation) were not removed from the study. Collected cycles in our study are in the range of 22–38 days with mean cycle duration of 28.87 ± 4.19 days (Table 1).

Although data was collected continuously, only the data during the subjects’ sleep was extracted to calculate daily values for each feature (Steps 1-3 in Figure 2) due to the inactivity of the subject and less motion artifacts (van Hees et al., 2018). Sleep hours were determined based on changes in the angle of the hand derived from accelerometry data: first calculated was the 5-min rolling median of the absolute differences between consecutive 5-s mean of the z-angle of data recorded during 8-pm to next day 10-am; then calculated was the 10th percentile of this data multiplied by 15 to define a threshold (van Hees et al., 2018); and finally the time of data less than this threshold specified sleep hours. Other criteria were also considered, including heart rate thresholds ( < 90 bpm) and sleep duration ( > 1-h). Lastly, daily average values were calculated from features extracted from data recorded within sleep hours.

In the next 2 steps (Step 4–5) cycles were interpolated (i.e., up/down sampled) to occupy a 28-day period to account for inconsistency in the cycle ranges. Data was normalized to the range of 1–10 after smoothing it by applying a causal moving average filter with length of 4. Finally, subjects’ cycles were averaged and converted from a linear scale to an angular scale (Steps 6–7).

### 2.5 Statistical analysis

To conduct statistical analysis, the phases of each cycle should be determined first. The menstrual cycle occurs in three main phases: follicular, ovulation, and luteal. Each interpolated 28-day cycle can be further classified into the five following phases: 1) menses (days 1–5), 2) mid-follicular phase (days 7–11), 3) ovulation phase (days 13–15), 4) mid-luteal phase (days 19–23) and 5) late-luteal phase (days 24–28). Menses was determined using the average menstruation duration listed in Table 1. The follicular phase is defined as the start of menstruation up until the day of ovulation (Fehring et al., 2006; Schmalenberger et al., 2021). Thus, the mid-follicular phase was determined to be 2 days post menstruation up to 2 days prior to ovulation. For an average 28-day cycle, the transition from the follicular phase to the luteal phase is known as ovulation and generally occurs on day 14 of the cycle (Prior et al., 2015). Therefore, the ovulation phase was defined to be days 13–15 to ensure inclusivity of the day of ovulation. The mid-luteal phase is characterized by constant low LH and FSH levels with elevated levels of progesterone (Figure 1B). Therefore, the mid-luteal phase was defined as days 19–23. Finally, the late-luteal phase starts when progesterone decreases and ends the day before menses (days 24–28). To show the significant difference between the different combinations of phases, a paired *t*-test was used.

**FIGURE 3:**
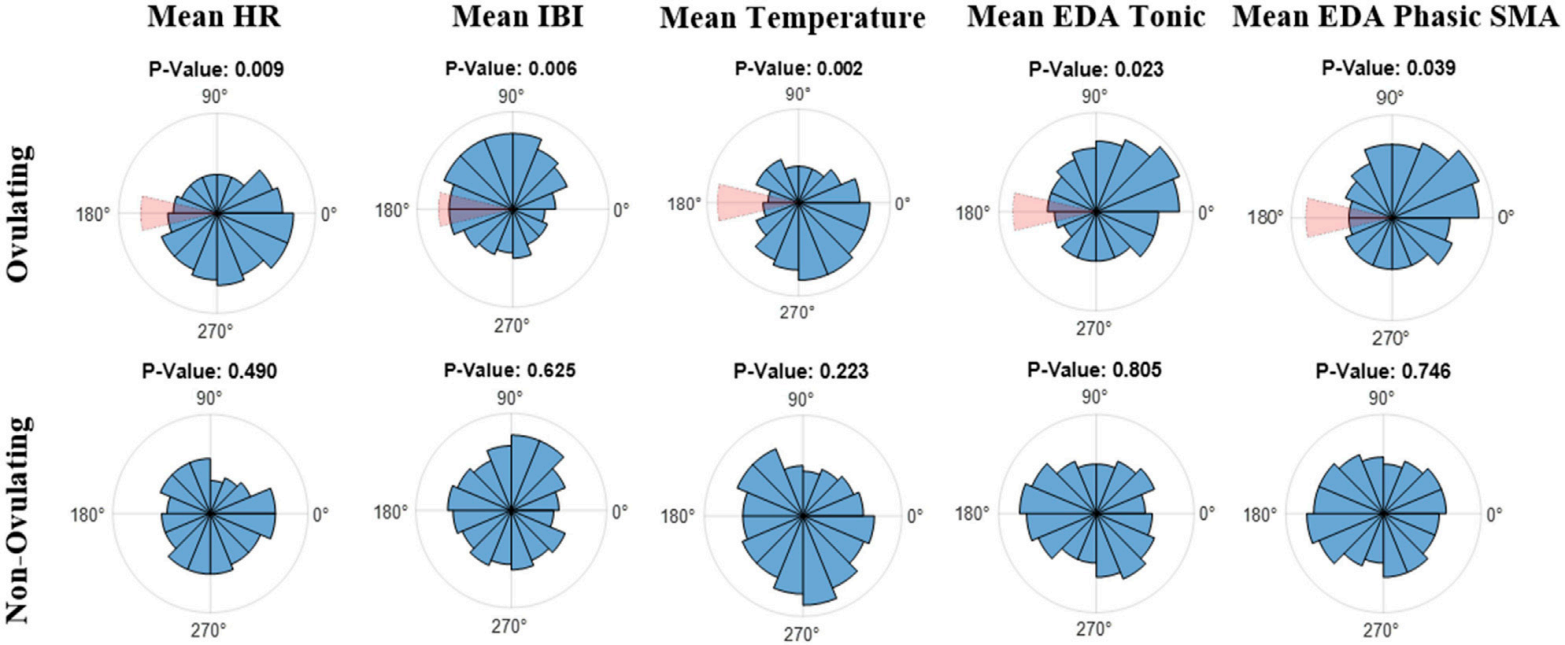
Polar Histogram plots (0°–359°) of the average values across the menstrual cycles for (top) ovulating, and (bottom) non-ovulating subjects; 31 ovulating cycles, 8 non-ovulating cycles. 0° indicates the start of menses and 180° indicates ovulation. Notice that the window of ovulation (days 13 through 15) is highlighted in red on polar histograms for ovulating cycles. The listed *p*-values were calculated using the Rayleigh test.

Lastly, data was mapped on an angular scale (0°–359°) using Matlab’s CircStat toolbox (Berens, 2009). In reference to the plots in Figure 3, 0° indicates the start of menses, or the first day of the subjects’ cycles, 180° marks ovulation, and 359° marks the end of the LH phase, and consequently the end of the 28-day cycle. Analyzed were both subject data on a per cycle basis as well as the combined average of all cycles. Figure 3 refers to the overall average of ovulating and non-ovulating cycles, respectively. The Rayleigh test was utilized for both ovulating and non-ovulating groups to detect the existence of a unimodal deviation from uniformity. A small p (p < 0.05) indicates a significant egress from uniformity and thus rejects the null hypothesis of uniform distribution (Berens, 2009). Rejections of the null hypothesis is an indication of a non-uniform pattern and thus represents ovulation. The Watson-Williams test, or the circular analogue of the two-sample *t*-test (Berens, 2009), was used to compare the two groups, namely, ovulating and non-ovulating cycles. This test assumes underlying von Mises distributions with equal concentration parameter and evaluates whether the mean directions of two or more groups are identical (Berens, 2009). Finally, to show the significant difference between the different combinations of phases, including menses, mid-follicular, ovulation, mid-luteal and late-luteal, a paired *t*-test was used.

### 2.6 Autoregressive integrated moving average (ARIMA) model

To predict physiological data for the following day, an autoregressive integrated moving average (ARIMA) algorithm was employed, which is well-suited for time series analysis. ARIMA models consist of three components: autoregressive (AR), integrated (I), and moving average (MA) terms. The AR term captures the relationship between a variable and its past values. The MA term captures the relationship between a variable and its past error terms, and the I part introduces differencing to make the time series stationary.

To determine the appropriate ARIMA model, first a moving average filter was applied to smooth out fluctuations, and then Augmented Dickey Fuller (ADF) test was used to confirm that data is stationarity (p < 0.05), thus the differencing parameter (known as *d*) was set to zero. To define the rest of the parameters the Autocorrelation Function (ACF), and Partial Autocorrelation Function (PACF) were used. Where the ACF measures the correlation between a time series and its lags at different intervals, and the PACF measures the correlation between a time series and its lags while controlling for shorter lags. Observing ACF and PACF plots (using training data for each subject individually), and after identifying significant spikes at certain lags (Kotu and Deshpande, 2018), the AR and MA terms for the ARIMA model was determined to be 2 and 3 (known as *p* and *q*). These values were selected since they were identifying significant spikes for majority of the subjects.

Here only the cycles with missing data (5 cycles) were removed which resulted in a total of 41 ovulating and non-ovulating cycles: more specifically, data included one subject with one cycle, 3 subjects with only 2 cycles each, 10 subjects with 3 cycles each and one subject with 4 cycles. Data from the subject with only one cycle was removed as well, resulting in 40 cycles. From mean values of temperature, HR, IBI and tonic EDA, the last cycle was kept for test while the rest (2 first cycles for 10 subjects, one first cycle for 3 subjects, and 3 first cycles for one subject) were used for initial training. It should be noted that each subject was fitted separately. The algorithm was re-trained every day by adding the new daily measure to training dataset, to predict the next day value. By analyzing patterns and trends in the data, the algorithm can identify potential changes and predict what is likely to happen in the next day.

## 3 Results

Using the Rayleigh test, in ovulating subjects, a non-uniform pattern was observed with a significant periodicity (p < 0.05) in mean temperature, HR, IBI, EDA tonic component, and SMA of the EDA phasic component in frequency domain among the extracted features reported in Section 2.2. Similar to the BBT chart in Figure 1A, there is a noticeable dip in amplitude of the polar histogram around 180° in the ovulating mean temperature chart in Figure 3 (top), indicating ovulation and representing a non-uniform distribution with a biphasic pattern (p < 0.05). The biphasic pattern was observed in mean EDA tonic and SMA of EDA phasic as well, although not as distinct as the mean temperature pattern. In contrast, non-ovulating cycles displayed a more uniform distribution in all selected features (p > 0.05).

Using the Watson-Williams test, a comparison was made between ovulating and non-ovulating groups. Unlike mean temperature, IBI and EDA, there was no significant difference (p > 0.05) between ovulating and non-ovulating cycle groups in mean HR (Table 2). The results of the tonic component were particularly interesting. Notice that in ovulating cycles (Figure 3), ovulation had a lower amplitude. In contrast, the associated window of ovulation for non-ovulating cycles had an elevated amplitude, albeit not significant compared to the rest of phases.

**TABLE 2:** Comparison of ovulating and non-ovulating cycles using the Watson-Williams test (Berens, 2009). This displays the differences between ovulating and non-ovulating averages from the following features: average HR, IBI, temperature, EDA skin conductance level, and SMA of the EDA phasic component in the frequency domain.

Feature	Test-statistic (F)	*p*-value
Mean Heart Rate	0.08495	0.770911343
Mean of IBI	5.10189	0.024588662
Mean Temperature	4.42631	0.036160184
EDA Signal Magnitude Area of Phasic Component	13.5693	0.000284669
Mean EDA Skin Conductance Level (Tonic Component)	20.6076	8.79E-06

**FIGURE 4:**
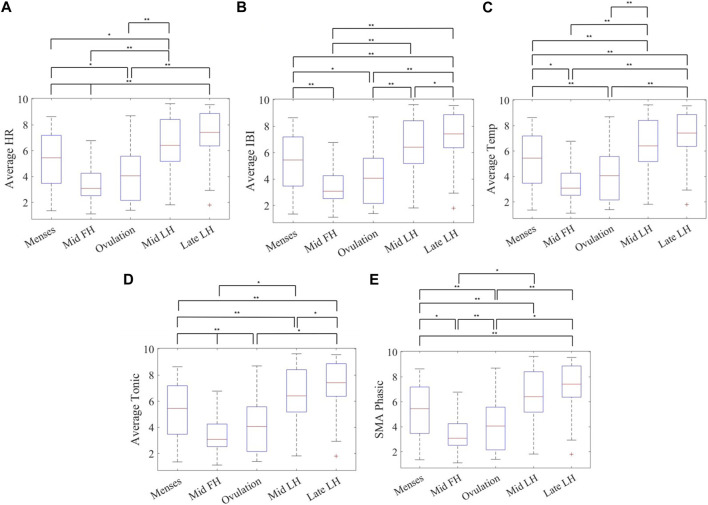
Mean value of phases across 31 cycles for **(A)** heart rate, **(B)** inter-beat-interval (IBI), **(C)** distal skin temperature, **(D)** tonic component of the EDA, and **(E)** and the EDA signal magnitude area of the phasic component in the frequency domain measured with the Empatica E4 wristband for ovulating subjects’ during sleep showing significant differences between five phases of the menstrual cycle using a paired *t*-test with a Bonferroni correction (*α* ≤ 0.005) (Phases: *Menses, Mid-follicular, Ovulation, Mid-luteal, and Late-luteal*) *n* = 31. **Note:** **α ≤ 0.005, *0.005 < *α* ≤ 0.05.

Comparison between combinations of phases including menses, mid-follicular, ovulation, mid-luteal and late-luteal, was performed using paired *t*-test after extracting the mean values of each phase from the smoothed and normalized data. In Figure 4, and outlined in Supplementary Table S1, ovulating cycles show significant differences between late-luteal phase and follicular phases (menses, mid-follicular) for mean temperature, HR and IBI as well as between late-luteal and menses for EDA mean tonic and SMA using a paired *t*-test with Bonferroni correction (*α* of 0.005). There is a significant difference between ovulation and mid-luteal phases in mean HR, IBI and temperature. No significant difference is observed between the mid-follicular phase and ovulation, except for EDA mean tonic and SMA.

Further, the ARIMA model was used to accurately predict the mean temperature, HR, IBI and EDA tonic of the following day (ovulating and non-ovulating), which resulted in the average root mean square error (RMSE) of 0.13 ± 0.07 (C°), 1.31 ± 0.34 (bpm), 0.016 ± 0.005 (s), and 0.17 ± 0.17 (*μ*S) respectively. Average RMSE across ovulating cycles for mean temperature, HR, IBI, and EDA tonic are 0.12 ± 0.03 (C°), 1.36 ± 0.35 (bpm), 0.017 ± 0.005 (s) and 0.15 ± 0.17 0.06 (*μ*S), respectively. The actual and predicated average daily values are shown in Supplementary Figure S1 for the third cycle of an ovulating subject with length of 26 days. The predicted curve is shown to follow the same pattern of the actual curve. These relatively low RMSE values show that ARIMA can be used to predict physiological signals based on previous data. This suggests wearable devices are a promising technology for predicting menstrual cycle phases using an appropriate ARIMA model.

## 4 Discussion

### 4.1 Principal findings

The purpose of the study was to implement circular statistics to investigate changes in physiological features during the natural menstrual cycle in women of reproductive age. Due to the periodic nature of the data used in this study, signals were converted to an angular scale and presented in degrees on a circular plot, which is different from commonly used linear techniques. On a circle 0° and 360° demonstrate the same direction, however, on a linear scale those points represent opposite ends of a scale, and much further apart (Cremers and Klugkist, 2018). Circular statistics gave us a tool to visualize the data in a different way.

Data from a wearable device (Empatica E4 wristband), combined with self-reported ovulation detection (LH-test strip) and a calendar with the mark of the start and end of menses were used to determine features representing significant phase shifts for ovulating cycles. Ovulating and non-ovulating cycles were also compared using an angular approach. Further, to determine the significance in deviation from uniformity in each feature, the Rayleigh test was used. The Rayleigh test was selected by observing the pattern in data; the distribution with one clear peak was observed in features after the occurrence of the nadir (lowest point) as in Figure 1A. However, the Rayleigh test generally assumes that the data is distributed normally (Karoly et al., 2018). To check uniformity, other statistical tests might also be considered such as the Rao’s spacing test, V test and Omnibus. Rao’s test was not a suitable option for this study because it is more applicable to data, that is, neither unimodal nor axially bimodal (Berens, 2009) and it did not confirm any significant phase shift in ovulating or non-ovulating subjects. The V test showed similar results as the Rayleigh test except for mean IBI. The V test for circular uniformity is similar to the Rayleigh test except that the mean direction of data has to be known before analyzing the data (Berens, 2009). The Omnibus test (Hodges-Ajne test) confirmed circular uniformity in ovulating subjects in mean temperature only. This test detects general deviations from uniformity but with less statistical power and without assumptions about the underlying distribution (Berens, 2009). In other words, the Omnibus test is used to check whether there is any circular pattern or non-random structure in the data. Although different tests were introduced to evaluate distribution uniformity, for unimodal distributions, the Rayleigh and V tests are more powerful than the Omnibus test, and among these two, the Rayleigh test is best recommended for unimodal departures from uniformity. However, in the multimodal case, the alternative tests should be considered (Landler et al., 2019). The Rayleigh test is suitable for unimodal distribution, but it might not be the best test to confirm biphasic pattern. Additionally, while both the Omnibus test and the Rayleigh test are used to assess the uniformity of circular data, the Omnibus test is more general and examines overall circular pattern, but the Rayleigh test is more focused on detecting unimodal distributions and the presence of specific orientations, or clustering, in circular data. It should be also noted that, although a biphasic pattern was observed in a few features such as mean temperature, it does not present a second significant peak to be considered as a bimodal distribution.

The findings demonstrated that wearable technology, with the ability to monitor multiple physiological features concurrently, including HR, IBI, temperature, and the EDA, can capture differences between ovulating and non-ovulating cycles. Of the many features extracted from the varying signals, only those with great significance in showing a non-uniform pattern regarding overall averages of cycles were reported. Many features had significance on a per subject, per cycle basis. One such feature, for example, was the power in 0.3–0.4 Hz band of the EDA phasic component. This feature, in particular, displayed significant phase shift in 10 out of the 12 ovulating subject averages. However, cycle averages indicated a more uniform distribution (p > 0.05). It was also interesting that a significant change was not observed across the menstrual cycle in LF and HF components of IBI despite findings in Vishrutha et al. (2012).

Additionally, the selected features were used in a regression model, specifically an Autoregressive Integrated Moving Average, to predict the physiological signals. It was shown that the ARIMA model accurately predicted the physiological values for the testing set, as evidenced by the relatively low value of the RMSE. Not a significant difference has been observed between ovulating and non-ovulating cycles in terms of RMSE. It is worth mentioning that data from non-ovulating cycles could be periodic but not biphasic. However, more non-ovulating cycles are needed to draw a clear conclusion.

The result from this study has many implications in reference to monitoring and predicting menstrual phases. For example, a reproductive-aged woman taking medication while trying to conceive would have the ability (along with medical advice from a licensed physician) to monitor medication dosages depending on the menstrual phase. Also, people with health issues that are linked to specific menstrual phase, such as catamenial epilepsy, can benefit from knowing if their cycle is ovulatory and accordingly adjust the anti-seizure medication dosage.

### 4.2 Limitations

There are several study limitations that may affect the accuracy of the wearable device. This includes, but is not limited to, device position, demographic factors and sleep assessment. Thus, the possibility that the Empatica E4 wristband may exhibit different levels of accuracy across different subjects, cycles and menstrual phases must be considered. A further limitation was that blood hormonal levels were not taken. This would have provided a more accurate method for detecting not only phases but ovulation itself. Although LH-tests (urine) were used, which are 99% accurate, there is potential for false negatives if the test was used incorrectly. A further limitation concerns the relatively small sample population size of non-ovulating cycles, especially compared to ovulating case (8 non-ovulating to 31 ovulating cycles). While we concluded the non-ovulating cycles exhibit a more uniform distribution, additional data has the potential to reveal that non-ovulating cycles also present a non-uniform distribution, but no less distinct from the non-uniform distribution of ovulating cycles. A larger sample size would, not only give better results, but accurately represent the two groups better.

## 5 Conclusion

In conclusion, angular methods can accurately represent the biphasic and non-uniform pattern in the data collected using a wearable device for naturally occurring menstrual cycles of reproductive-aged women. There is significant difference in ovulating and non-ovulating cycles. These results can fuel algorithms, such as ARIMA, to accurately predict human physiological signals and potentially classify menstrual phases via machine learning algorithms. Thus, wearable technology is a promising tool for tracking physiological changes across the menstrual cycle.

## Data Availability

The original contributions presented in the study are included in the article/Supplementary Material, further inquiries can be directed to the corresponding author.
