# Supplementary material for: Analyzing physiological signals recorded with a wearable sensor across the menstrual cycle using circular statistics
Source: Front Netw Physiol. 2023 Oct 19;3:1227228. doi: 10.3389/fnetp.2023.1227228 (PMC10621043; doi:10.3389/fnetp.2023.1227228)
Supplement: Supplementary file 1 [file Presentation1.zip › Supplementary Table 1 & Figure 1.PDF]

Supplementary Material

1 SUPPLEMENTARY DATA

1.1 Figures

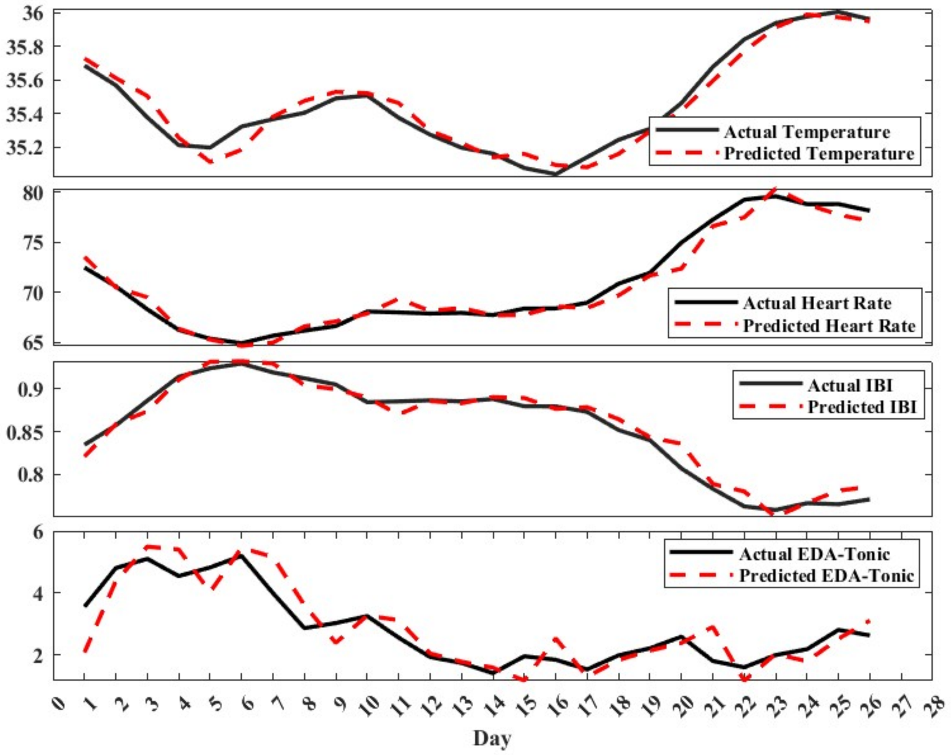

Figure S1. The actual and ARIMA predicated average daily values for third cycle of an ovulating subject with length of 26 days with RMSE values of 0.063 (C°), 0.89 (bpm), 0.011 (s), and 0.6 (μS).

1.2 Tables

Average Heart Rate

Phase	*p-value*
Menses & Mid FH	**2.388E-04
Menses & OV	*0.022
Menses & Mid LH	*0.024
Menses & Late LH	**1.689E-04
Mid FH & OV	0.143
Mid FH & Mid LH	**1.486E-08
Mid FH & Late LH	**4.974E-12
OV & Mid LH	**1.036E-05
OV & Late LH	**8.328E-09
Mid LH & Late LH	0.114

Table S1. Results for significant changes of average heart rate across 5 phases of the menstrual cycle (*Menses, Mid-follicular, Ovulation, Midluteal, and Late luteal*), $n = 31$ cycles using a paired t-test with a Bonferroni correction. **Note:** **$\alpha \leq 0.005$, *$0.05 \geq \alpha > 0.005$.

Average Inter-beat Interval

Phase	*p-value*
Menses & Mid FH	**3.309E-05
Menses & OV	*0.009
Menses & Mid LH	0.054
Menses & Late LH	**5.325E-05
Mid FH & OV	0.101
Mid FH & Mid LH	**6.203E-09
Mid FH & Late LH	**5.951E-14
OV & Mid LH	**1.031E-05
OV & Late LH	**3.343E-10
Mid LH & Late LH	*0.027

Table S2. Same as table S1 except for average inter-beat-interval. **Note:** **$\alpha \leq 0.005$, *$0.05 \geq \alpha > 0.005$.

Average Temperature

Phase	*p-value*
Menses & Mid FH	*0.013
Menses & OV	**0.003
Menses & Mid LH	**1.779E-06
Menses & Late LH	**6.360E-09
Mid FH & OV	0.644
Mid FH & Mid LH	**9.809E-12
Mid FH & Late LH	**1.421E-14
OV & Mid LH	**8.615E-13
OV & Late LH	**1.110E-15
Mid LH & Late LH	0.221

Table S3. Same as table S1 except for average temperature. **Note:** **$\alpha \leq 0.005$, *$0.05 \geq \alpha > 0.005$.

Average EDA Tonic Component

Phase	*p-value*
Menses & Mid FH	**0.001
Menses & OV	**1.401E-08
Menses & Mid LH	**4.132E-08
Menses & Late LH	**3.009E-04
Mid FH & OV	**0.005
Mid FH & Mid LH	*0.010
Mid FH & Late LH	0.640
OV & Mid LH	0.819
OV & Late LH	*0.020
Mid LH & Late LH	*0.035

Table S4. Same as table S1 except for average EDA tonic component. **Note:** **$\alpha \leq 0.005$, *$0.05 \geq \alpha > 0.005$.

Average SMA of the Phasic Component

Phase	*p-value*
Menses & Mid FH	*0.007
Menses & OV	**1.221E-08
Menses & Mid LH	**9.306E-07
Menses & Late LH	**4.306E-04
Mid FH & OV	**0.001
Mid FH & Mid LH	*0.017
Mid FH & Late LH	0.384
OV & Mid LH	0.347
OV & Late LH	*0.014
Mid LH & Late LH	0.123

Table S5. Same as table S1 except for the EDA signal magnitude area of the phasic component. **Note:** **$\alpha \leq 0.005$, *$0.05 \geq \alpha > 0.005$.
